# Is the sarcomatous component (homologous vs heterologous) the prognostic “driving force” in early-stage uterine carcinosarcomas? A retrospective multicenter study

**DOI:** 10.1007/s00432-023-04594-5

**Published:** 2023-02-11

**Authors:** A. Rosati, V. Vargiu, C. Certelli, M. Arcieri, E. Vizza, F. Legge, F. Cosentino, G. Ferrandina, F. Fanfani, G. Scambia, G. Corrado

**Affiliations:** 1 Dipartimento Scienze della Salute della Donna, del Bambino, e di Sanità Pubblica, Ginecologia Oncologica, Fondazione Policlinico Universitario A. Gemelli IRCCS, L.go A. Gemelli 8, 00168 Rome, Italy; 2 Department of Gynecologic Oncology, Gemelli Molise, Campobasso, Italy; 3 Department of Experimental Clinical Oncology, Gynecologic Oncology Unit, IRCCS “Regina Elena” National Cancer Institute, Rome, Italy; 4 Gynecologic Oncology Unit, Dept. Obstetrics/Gynecology “F. Miulli” General Regional Hospital, Acquaviva delle Fonti, Bari, Italy; 5 Department of Gynecologic Oncology, Gemelli Molise, Campobasso, Italy; 6 Università Cattolica del Sacro Cuore, Rome, Italia; 7 Dipartimento di Medicina e Scienze della Salute “Vincenzo Tiberio” Università degli studi del Molise, Campobasso, Italy

**Keywords:** Uterine carcinosarcomas, Malignant mixed mullerian tumors, Homologous component, Heterologous component, Sarcomatous component

## Abstract

**Purpose:**

Uterine carcinosarcomas (UCSs) are aggressive biphasic malignancies, with a carcinomatous/epithelial component and a sarcomatous/mesenchymal counterpart. The aim of this study was to evaluate the impact of the sarcomatous component (homologous vs heterologous) on the overall survival (OS) and progression-free survival (PFS).

**Methods:**

This is a multicenter observational retrospective study conducted in patients with stage I and II UCSs.

**Results:**

Ninety-five women with histological diagnosis of early-stage UCSs were retrieved: 60 (63.2%) had tumors with homologous sarcomatous components, and 35 (36.8%) with heterologous. At univariate analysis, a stromal invasion ≥ 50%, the presence of clear cell, serous or undifferentiated carcinomatous component, the heterologous sarcomatous component and FIGO stage IB and II were shown to be variables with a statistically significant negative impact on PFS. Similarly, a depth of invasion ≥ 50%, the heterologous sarcomatous component and FIGO stage IB and II were statistically negative prognostic factors also concerning OS. At multivariate analysis, only the heterologous sarcomatous component was confirmed to be a statistically significant negative prognostic factor both on PFS (HR 2.362, 95% CI 1.207–4.623, *p* value = 0.012) and on OS (HR 1.950, 95% CI 1.032–3.684, *p* = 0.040).

**Conclusion:**

Carcinomatous and sarcomatous components both played a role in tumor progression and patients’ survival. However, only the sarcomatous component retained a statistical significance at the multivariable model suggesting its preeminent prognostic role in early-stage UCSs.

**Supplementary Information:**

The online version contains supplementary material available at 10.1007/s00432-023-04594-5.

## Introduction

Uterine carcinosarcomas (UCSs), otherwise referred as malignant mixed mullerian tumors (MMMT), are rare and aggressive biphasic malignancies (Cantrell et al. 2015; Bansal et al. 2008; Hosh et al. 2016), composed by a carcinomatous/epithelial component and a sarcomatous/mesenchymal counterpart (Matsuzaki et al. 2021).

The two most accepted histopathogenic hypotheses are the “multiclonal”, in which the two components evolved independently from two distinct cellular origins, and the “monoclonal” one, which, on the contrary, suggests that UCSs derive from a common multipotent stem cell that differentiates in the two components during tumorigenesis (Ng et al. 2003; Jin et al. 2003; Mccluggage 2002).

The activation of a stable epithelial to mesenchymal transition (EMT) represents the background of the monoclonal theory in which the sarcomatous elements would derive from the de-differentiation of the carcinomatous one (Castilla et al. 2011; Wada et al. 1997). The carcinoma component has, therefore, been shown to be the pathogenetic "driving force" in UCSs both immunohistochemically and molecularly (McCluggage 2002).

According to this, UCSs have been reallocated in the classification of endometrial carcinomas (McCluggage 2002; Concin et al. 2021 Jan), although they appear to be biologically different from other high-risk histologies with an extremely poor prognosis even in early-stage tumors (George et al. 1995; Vaidya et al. 2006; Amant et al. 2005).

This argues that sarcomatous elements, within an endometrial carcinoma, may be the prognostic keystone of an increased biological aggressiveness (George et al. 1995).

However, which of the two histological components has the greatest prognostic impact is still one of the most debated topics in the literature, with several studies “favoring” the carcinomatous component (Nordal et al. 1997; Iwasa et al. 1998; Silverberg et al. 1990), and others the sarcomatous one (Ferguson et al. 2007; Makker et al. 2008). Furthermore, the limited success of adjuvant therapy suggest that considerable progress still needs to be made in understanding the molecular biology of this subclass of tumors in order to tailor the most appropriate therapeutic strategy.

The aim of our study was to evaluate the impact of tumor histological characteristics on disease-free survival (DFS) and overall survival (OS) in a large cohort of early-stage UCSs (FIGO stage I-II).

In addition, we evaluated the differences in terms of clinical and pathological characteristics between homologous and heterologous tumors and specifically assessed the impact of the sarcomatous component (homologous vs heterologous) on oncological outcomes.

## Materials and methods

This is a multicenter observational retrospective study conducted in patients with early-stage UCS evaluated at four referral cancer centers in Italy (Fondazione Policlinico Universitario A. Gemelli, IRCCS, Rome; Istituto Regina Elena, Rome; Regional General Hospital “F. Miulli”, Acquaviva delle Fonti, Bari, and Gemelli Molise S.p.A, Campobasso).

This study was approved by the internal review board.

An informed consent was signed by all women for their data to be collected and analyzed for scientific purpose.

All consecutive patients with histological diagnosis of UCS from May 2003 to March 2019 were selected and evaluated for the analysis.

Inclusion criteria were histological diagnosis of UCS confined to the uterus (FIGO stage I and II), age of 18 years old or older, clinical performance status 0–2 (Eastern Cooperative Oncologic Group-ECOG).

Patients who were upstaged at the final pathological examination and patients with diagnosis of epithelial endometrial cancer or pure uterine sarcoma were excluded from the study.

The study population was first analyzed in its entirety and then divided in two groups according to the presence of homologous or heterologous sarcomatous component.

The homologous sarcomatous subtype included the endometrial stromal sarcomas and the leiomyosarcomas, while the heterologous sub-group was represented by a sarcomatous component mimicking ectopic tissues, i.e., chondrosarcomas, osteosarcomas, rhabdomyosarcomas and liposarcomas (Jin et al. 2003).

The following clinical and surgical data were collected: age, body mass index (BMI), presence of comorbidity (i.g. cardiovascular, respiratory, renal, gastroenterological disease), surgical approach (laparoscopic or laparotomic), type of radical hysterectomy according to the Querleu-Morrow classification (type A, B, C), omental and nodal status assessment (none, pelvic, pelvic plus lumbo-aortic), operative time and the intra-operative estimated blood loss (EBL).

Details regarding the histopathological examinations were retrieved and analyzed (FIGO stage—categorized into stage IA, IB and II; histologic subtype of epithelial component—endometrioid G1/G2, endometrioid G3, serous high-grade/clear cell/undifferentiated; maximum tumor diameter < 50 mm or ≥ 50 mm; depth of invasion < 50% or ≥ 50%; presence or absence of LVSI).

Data about postoperative complications and details referring to the administration of adjuvant treatment and survival outcomes were also recovered.

### Statistical analysis

Descriptive statistics was used to describe the patient, surgical, and pathological characteristics.

Qualitative variables have been summarized as absolute and percentage frequencies, quantitative variables have been summarized by their median and interquartile range (IQR).

The normality of data has been verified via the Kolmogorov–Smirnov test.

Groups were compared using the Mann–Whitney *U* test for continuous variables and the Pearson *χ*^2^ test (or Fisher exact test when required) for categorical variables.

Progression-free survival (PFS) was calculated from the diagnosis (hysteroscopic or surgical) until the disease progression or the last follow-up.

Overall survival (OS) was calculated from the diagnosis to death or the date last seen.

Survival outcomes were assessed according to a series of potentially prognostic histopathological factors using both the Kaplan–Meier and Cox regression models. Hazard ratios (HRs) and 95% confidence intervals (CIs) were calculated for each comparison and univariate and multivariate analyses were performed when appropriate. All statistical tests were two-sided and a value of *p* < 0.05 was considered as statistically significant.

Statistical calculations were performed using the Statistical Package for Social Sciences (Version 27; SPSS Inc., Chicago, IL).

## Results

### Patients and surgical data

Data from 95 women with histological diagnosis of early-stage UCS (FIGO stage IA-II) were retrieved.

Of these, 60 (63.2%) had tumors with homologous sarcomatous components (Group1), and 35 (36.8%) had tumors classified as heterologous (Group2).

Patients’ clinical and surgical characteristics are shown in Table 1.

**Table 1:** Baseline and surgical characteristics

Baseline characteristics				
Variables	All *N* (%) *N* = 95	Sarcomatous component		*p* value^†^
		Homologous/Group1 *N* (%) *N* = 60	Heterologous/Group2 *N* (%) *N* = 35	
Age*	68.0 (62.0–75.0)	65.00 (60.25–73.00)	71 (65.00–80.00)	**0.008**^**‡**^
BMI*	28.0 (25.0–30.0)	28.00 (25.00–30.00)	28 (24.00–30.00)	0.855
Comorbidity				**0.010**
None	22 (23.2)	19 (31.7)	3 (8.6)	
Yes	73 (76.8)	41 (68.3)	32 (91.4)	
Surgical characteristics				

Variables	All *N* (%) *N* = 95	Sarcomatous component		*p* value^†^
		Homologous/Group1 *N* (%) *N* = 60	Heterologous/Group2 *N* (%) *N* = 35	
Surgical approach				0.606
LPS	51 (53.7)	31 (51.7)	20 (57.1)	
LPT	44 (46.3)	29 (48.3)	15 (42.9)	
Radical hysterectomy**				0.999 fisher
A	83 (87.4)	52 (86.7)	31 (88.6)	
B	12 (12.6)	8 (13.3)	4 (4.2)	
Omental biopsy/omentectomy				0.893
No	74 (77.9)	47 (78.3)	27 (77.1)	
Yes	21 (22.1)	13 (21.7)	8 (22.9)	
Nodal status assessment				**0.035**
None	38 (40.0)	23 (38.3)	15 (42.9)	
Pelvic sampling/lymphadenectomy	45 (47.4)	33 (55.0)	12 (34.3)	
LA sampling/lymphadenectomy	12 (12.6)	4 (6.7)	8 (22.9)	
Operative time* (min)	150.0 (120.0–210.0)	147.50 (120.00–198.75)	150.00 (100.00–220.00)	0.688
EBL* (mL)	100.0 (50.0–200.0)	100.00 (50.00–200.00)	100.00 (50.00–200.00)	0.481

Bold values: *p* value < 0.05

*BMI* body mass index, *LPS* laparoscopy, *LPT* laparotomy, *SLN* sentinel lymph node, *EBL* estimated blood loss, *LA* lomboaortic

*Median (interquartile range)

**According to Querleu-Morrow classification

^**‡**^*U* Mann–Whitney test

^†^*χ*-squared test

The median age of the study population was 68 years, but stratifying patients on the sarcomatous component (homologous vs heterologous), patients with heterologous tumors were significantly older and with a higher preoperative comorbidity rate than the homologous ones (Group1 vs Group2: median age 65.00 vs 71.00, *p* value = 0.008, comorbidity rate 68.3 vs 91.4%, *p* value = 0.010).

Concerning surgical characteristics (Table 1), the two groups only differed in terms of nodal assessment. Pelvic and lombo-aortic lymphadenectomy or sampling were more commonly performed in patients with heterologous component (*p* value = 0.035).

In the whole series, 38 patients did not receive nodal staging procedures because of age or comorbidities.

Table 2 shows in detail the histopathological features of the study population.

**Table 2:** Histological features

Variables		Sarcomatous component		*p* value^†^
	All *N* (%) *N* = 95	Homologous/Group1 *N* (%) *N* = 60	Heterologous/Group2 *N* (%) *N* = 35	
FIGO stage				0.200
IA	38 (40.0)	28 (46.7)	10 (28.6)	
IB	36 (37.9)	21 (35.0)	15 (42.9)	
II	21 (22.1)	11 (18.3)	10 (28.6)	
Epithelial part				0.076
Endometrioid G1-G2	19 (22.1)	15 (28.8)	4 (11.8)	
Endometrioid G3	37 (43.0)	23 (44.2)	14 (41.2)	
Other (serous/clear cell/undifferentiated)	30 (34.9)	14 (27.0)	16 (47.0)	
NA	9	8	1	
Maximum tumor diameter				**0.002**
< 50 mm	35 (36.8)	29 (48.3)	6 (17.1)	
≥ 50 mm	60 (63.2)	31 (51.7)	29 (82.9)	
Depth of invasion				0.078
< 50%	41 (43.2)	30 (50.0)	11 (31.4)	
≥ 50%	54 (56.8)	30 (50.0)	24 (68.6)	
LVSI				**0.001**
Absent	53 (59.6)	40 (74.1)	13 (37.1)	
Present	36 (40.4)	14 (25.9)	22 (62.9)	
NA	6			

Bold values: *p* value < 0.05

*FIGO* International Federation of Gynecology and Obstetrics, *NA* not available, *LVSI* lymph-vascular space invasion

^†^*χ*-squared test

Tumors with sarcomatous heterologous component were significantly larger than their homologous counterpart (Group2 and Group1 T ≥ 50 mm: 82.9 vs 51.7%, *p* value = 0.002) and presented more frequently lymph-vascular space invasion (62.9 vs 25.9%, respectively, in Group2 and Group1, *p* value = 0.001).

Anyhow, the two groups did not differ in terms of FIGO stage and depth of myometrial invasion (*p* value = 0.200 and *p* value = 0.078, respectively).

The epithelial components of UCSs was serous/clear cell/undifferentiated in 34.9% of cases (30/95), endometrioid G3 in 43% (37/95), while less frequently endometrioid G1–G2 (22.1%, 19 out of 95 patients).

A different trend in the distribution of the epithelial components has been identified between Group 1 and 2 (*p* value = 0.076), in fact, heterologous tumors were associated to endometrioid G1–G2 epithelial subtype in only 11.8% of cases (4/35), to endometrioid G3 in 41.2% of cases (14/35) and in 47.0% of cases to a serous/clear cell/undifferentiated epithelial component (16/25). Conversely the reported rates in the “homologous group” were of 28.8%, 44.2% and 27.0%, respectively.

### Post-operative features and survival outcomes

Early postoperative outcomes and adjuvant treatment were comparable in the two groups (Table S1).

Considering the whole population, 83.2% of patients received adjuvant treatment, which consisted of EBRT/brachytherapy (17.7%), chemotherapy alone (39.2%) or the combination of the two (43.0%).

Sixteen patients did not receive any adjuvant treatment (16.8%) due to advanced age (> 80 years), patient’s refusal or decay of the clinical condition.

Regarding survival outcomes, 50 out of 95 patients (52.6%) experienced recurrence, while 42 patients (44.2%) died of disease.

The most frequent recurrence site was pelvic (44% of relapses) with 12 cases in the heterologous group and 10 in the homologous group. We then observed 15 cases of distant metastases (4 in the heterologous group and 11 in the homologous group) and 7 lymph node metastases (5 and 2 respectively). The remaining relapses were classified as mixed (five in the heterologous group and one in the homologous group).

**Fig. 1:**
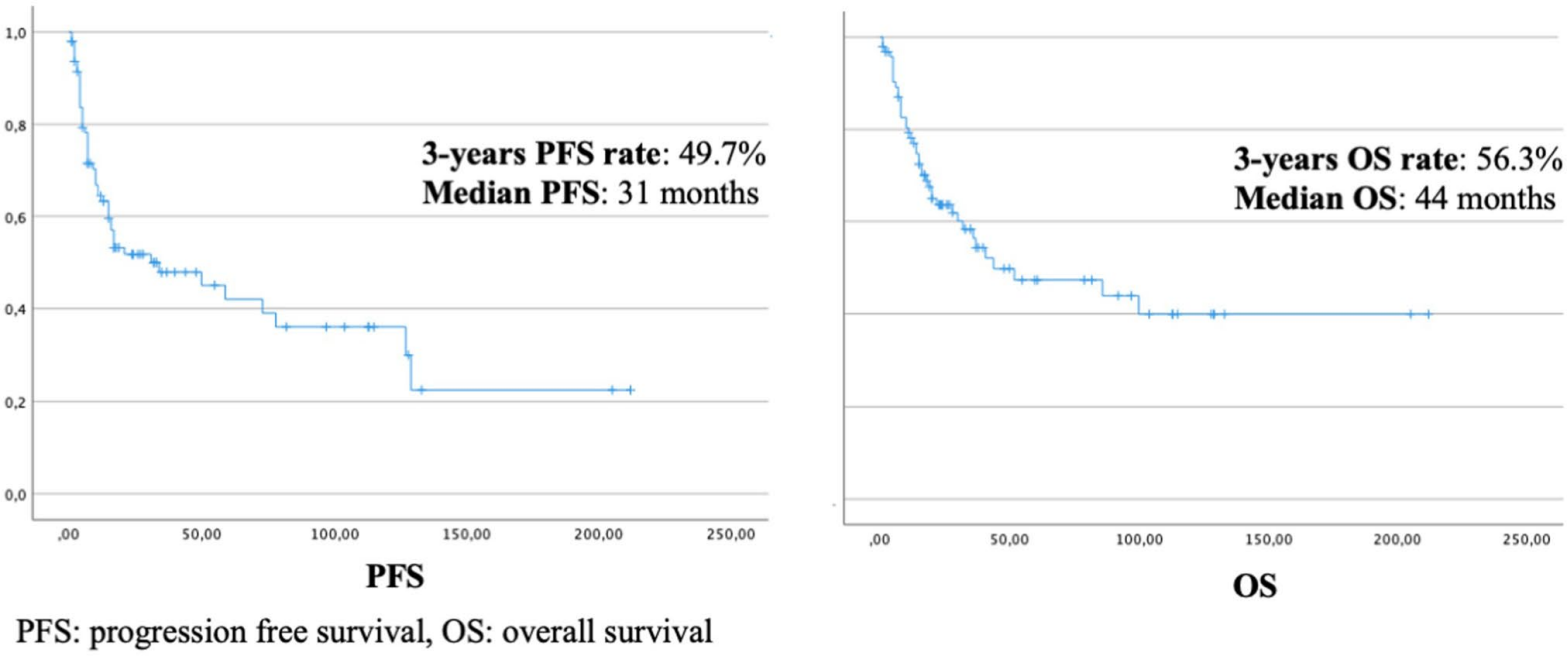
Kaplan–Meier plots for progression-free survival and overall survival

In the whole series, the 3 years PFS and OS were 49.7% and 53.6% (median PFS 31 months, median OS 44 months) (Fig. 1).

At univariate analysis, a stromal invasion ≥ 50%, the presence of clear cell, serous or undifferentiated carcinomatous component, the heterologous sarcomatous component and the FIGO stage IB and II demonstrated a statistically significant negative impact on PFS (depth of invasion ≥ 50% HR 2.811, 95% CI 1.507–5.244, *p* value = 0.001; clear cell/serous/undifferentiated epithelial component HR 2.405, 95% CI 1.009–5.733, *p* value = 0.048; heterologous sarcomatous component subtype HR 3.220, 95% CI 1.771–5.857, *p* value < 0.001; FIGO stage IB HR 2.437, 95% CI 1.183–5.020, *p* value = 0.016 and FIGO stage II HR 4.431, 95% CI 2.114–9.289, *p* value < 0.001).

Nevertheless, at multivariate analysis, only the heterologous sarcomatous component was confirmed as a statistically relevant negative prognostic factor (HR 2.362, 95% CI 1.207–4.623, *p* value = 0.012) (Table 3).

**Table 3:** Cox regression analysis of clinicopathologic factors for progression-free survival in women with early-stage UCS: univariate and multivariate analysis

Variables	Univariate analysis		Multivariate analysis	
	HR (95% CI)	*p* value	HR (95% CI)	*p* value
Maximum tumor diameter			–	–
< 5 cm	1			
≥ 5 cm	1.407 (0.768–2.580)	0.269		
Depth of myometrial invasion				
< 50%	1		1	
≥ 50%	**2.811 (1.507–5.244)**	**0.001**	1.288 (0.282–5.886)	0.744
LVSI			–	–
Absent	1			
Present	1.635 (0.905–2.953)	0.103		
Histologic subtype of epithelial component				
Endometrioid G1-2	1		1	
Endometrioid G3	1.563 (0.694–3.521)	0.281	1.226 (0.540–2.784)	0.627
Other (serous/clear cell/undifferentiated)	**2.405 (1.009–5.733)**	**0.048**	1.746 (0.693–4.400)	0.237
Histologic subtype of sarcomatous component				
Homologous	1		1	
Heterologous	**3.220 (1.771–5.857)**	** < 0.001**	**2.362 (1.207–4.623)**	**0.012**
FIGO stage				
IA	1		1	
IB	**2.437 (1.183–5.020)**	**0.016**	1.975 (0.363–10.752)	0.431
II	**4.431 (2.114–9.289)**	** < 0.001**	3.137 (0.660–14.912)	0.151

Bold values: *p* value < 0.05

Similarly, a depth of invasion ≥ 50%, the heterologous sarcomatous component and the FIGO stage IB and II were statistically negative prognostic factors also concerning OS (depth of invasion ≥ 50% HR 2.888, 95% CI 1.447–5.762, *p* = 0.003; heterologous sarcomatous component HR 2.484, 95% CI 1.335–4.621, *p* = 0.004, FIGO stage IB HR 2.460, 95% CI 1.122–5.394, *p* = 0.025, FIGO stage II HR 3.709, 95% CI 1.659–8.291, *p* = 0.001), but even in this case, only the heterologous sarcomatous component maintained its negative independent prognostic value (HR 1.950, 95% CI 1.032–3.684, *p* = 0.040) (Table 4).

**Table 4:** Cox regression analysis of clinicopathologic factors for overall survival in women with early-stage UCS: univariate and multivariate analysis

Variables	Univariate analysis		Multivariate analysis	
	HR (95% CI)	*p* value	HR (95% CI)	*p* value
Maximum tumor diameter			–	–
< 5 cm	1			
≥ 5 cm	1.688 (0.847–3.364)	0.137		
Depth of invasion				
< 50%	1		1	
≥ 50%	**2.888 (1.447–5.762)**	**0.003**	2.132 (0.276–16.482)	0.468
LVSI			-	-
Absent	1			
Present	1.427 (0.746–2.730)	0.282		
Histologic subtype of epithelial component			–	–
Endometrioid G1-G2	1			
Endometrioid G3	1.468 (0.578–3.725)	0.419		
Other (serous/clear cell/undifferentiated)	1.934 (0.728–5.141)	0.186		
Histologic subtype of sarcomatous component				
Homologus	1		1	
Heterologus	**2.484 (1.335–4.621)**	**0.004**	**1.950 (1.032–3.684)**	**0.040**
FIGO Stage				
IA	1		1	
IB	**2.460 (1.122–5.394)**	**0.025**	1.053 (0.118–9.415)	0.963
II	**3.709 (1.659–8.291)**	**0.001**	1.543 (0.195–12.199)	0.681

Bold values: *p* value < 0.05

*UCS* uterine carcinosarcoma, *HR* hazard ration, *CI* confidence interval, *LVSI* lymph-vascular space invasion, *FIGO* International Federation of Gynecology and Obstetrics

**Fig. 2:**
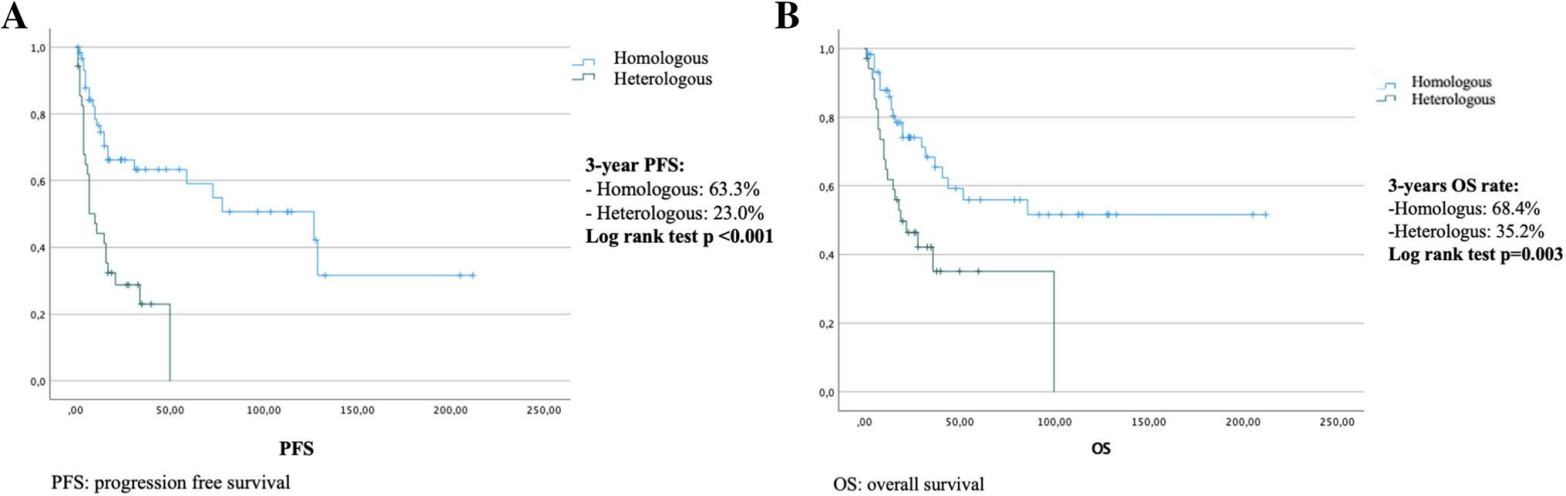
Kaplan–Meier plots for progression-free survival and overall survival according to the histological sarcomatous subtype component (**A** progression-free survival, **B** overall survival)

Analyzing the prognostic impact of the sarcomatous component, we found a 3-year PFS rates of 63.3% and 23.0%, respectively, in the homologous and heterologous group (log rank test: *p* value < 0.001) (Fig. 2A), while the 3-year OS rates were 68.4% and 35.3% (log rank test: *p* value = 0.003) (Fig. 2B).

In a sub-analysis that stratified patients for lymph node assessment into homologous and heterologous groups, we found a statistically significant difference in prognosis in both PFS and OS (log rank test, respectively: *p* value 0.008 and *p* value < 0.001) (Fig. 1S). This finding supports the crucial importance of lymph node staging in uterine carcinosarcoma and its prognostic relevance in both groups.

Furthermore, although the epithelial component was not found to be a statistically significant prognostic factor on the multivariable model, this is potentially due to the paucity of data.

Indeed, we have to acknowledge that the low-grade endometrioid component (G1-2 endometrioid) seems to represent a positive prognostic factor in both PFS and OS (log rank test, respectively: *p* value < 0.001 and *p* value-0.011) (Fig. 2S) compared with high-grade, serous, clear cell, undifferentiated endometrioid histology in homologous carcinosarcomas.

## Discussion

### Summary of main results

Although the type-2 epithelial histotypes correlated with an increased risk of relapse at univariate analysis, only the heterologous sarcomatous component was confirmed as an independent risk factor for both PFS and OS on a multivariable model.

In addition, UCSs with a heterologous component showed larger dimensions, a higher rate of LVSI and occurred in older patients than their homologous counterpart.

### Results in the context of literature

UCSs are very aggressive and rare disease with an unfavorable prognosis, indeed, even considering only FIGO stage I–II, the 3-year PFS and OS rates were still relatively low (49.7% and 56.3%), confirming what has already been reported in the literature (Bansal et al. 2008; Hosh et al. 2016; Amant et al. 2005; Gonzalez Bosquet et al. 2010; Gadducci et al. 2002; El-Nashar and Mariani 2011).

FIGO stage is actually considered the main and most reliable prognostic factor (Hosh et al. 2016; Amant et al. 2005; Gonzalez Bosquet et al. 2010; Callister et al. 2004).

However, given the poor prognostic outcomes, the identification of other possible histological prognostic predictors would be advisable, to better tailor peri and postoperative management and to identify the appropriate adjuvant therapy (Corrado et al. 2021).

Indeed, guidelines are currently extremely vague, mainly based on the FIGO stage (Concin et al. 2021; Network and Guidelines 2021) and neglecting the extreme histological heterogeneity of the UCSs.

There are conflicting data in literature whether the prognostic “driving force” is represented by the epithelial or the sarcomatous component (George et al. 1995; Iwasa et al. 1998; Silverberg et al. 1990; Matsuo et al. 2016; Chen et al. 2017), furthermore, there are various possible combinations between the two parts.

As reported by Matsuo et al. (2016) the most frequent epithelial/sarcomatous association was the high-grade/homologous (which included grade 3 endometrioid, serous, clear cell, undifferentiated, and mixed histology subtypes), followed by high-grade/heterologous, low-grade/homologous, and lastly low-grade/heterologous.

In our series, an even stronger association between type-2 epithelial histotype (serous, clear cell, undifferentiated) and heterologous sarcomatous component was reported, while the association between endometrioid low-grade/heterologous represented just a minority of cases (11.8% low-grade/heterologous vs 28.8% of low-grade/homologous)); with advances in molecular profiling in the future we will potentially be able to elucidate the molecular landscape of this association between more “aggressive” epithelial histotypes and the heterologous sarcomatous component.

Given the most accredited "carcinoma-leader" hypothesis, many authors have argued that a more aggressive epithelial component, i.e. serous, clear-celled or undifferentiated, represents the most relevant prognostic histopathological feature (Nordal et al. 1997; Silverberg et al. 1990; Matsuo et al. 2016; Leath et al. 2009).

Conversely, other authors found that the carcinoma component did not affect survival outcomes (Chen et al. 2017; Harano et al. 2016; Sartori et al. 1997), attributing a greater prognostic relevance to the sarcoma dominance or to sarcoma grade and differentiation (Ferguson et al. 2007; Major et al. 1993; Abdulfatah et al. 2019; Kurnit et al. 2019). Specifically, examining histopathological variables at Cox multivariate analysis, Abdulfatah et al. (2019) identified the sarcoma dominance as a significant factor for decreased DFS (HR 2.45, 95% CI 1.21–4.94, *p* = 0.012) and the rhabdomyoblastic differentiation as independent negative predictor for OS (HR 2.73, 95% CI 1.28–5.81, *p* = 0.009). The presence of rhabdomyosarcoma component was confirmed as an independent risk factor for OS even by Kurnit et al. (HR of 1.66, *p* = 0.041. In line with previous studies (Matsuo et al. 2016; Abdulfatah et al. 2019; Kurnit et al. 2019; Dinh et al. 1989), we also identified, at univariate analysis, deep myometrial invasion, heterologous sarcomatous component and FIGO stage as negative prognostic factors both for PFS and OS.

However, according to Abdulfatah et al. (2019) and Kurnit et al. (2019), on Cox multivariable model, the only independent negative prognostic factor for both PFS and OS was the presence of heterologous sarcomatous component with a twofold risk of recurrence and death compared to the homologous one (PFS HR: 2.362, 95% CI 1.207–4.623, *p* = 0.012 and OS HR: 1.950, 95% CI 1.032–3.684, *p* = 0.040).

The heterologous sarcomatous component represented only a minority of an already rare disease (33.4%). Other differences that suggest the greater biological aggressiveness of the heterologous UCSs over the homologous ones, were their larger size, as already reported by Kurnit et al. (2019), and the higher frequency of LVSI. These results should lead us to reconsider the central role of the sarcomatous component for the modulation of the follow-up schedule and the adjuvant therapy.

Although both components seem to have the same origin, they showed different dissemination and recurrence patterns, i.e. lymphotropic for the epithelial component and loco-regional or hematogenous for the sarcomatous (Amant et al. 2005; Matsuo et al. 2016; Kernochan and Garcia 2009).

As previously anticipated, NCCN (2021) and ESMO ESGO ESTRO (Concin et al. 2021) guidelines propose an adjuvant treatment with carboplatin paclitaxel plus EBRT for UCSs depending on the FIGO stage but irrespective of their intrinsic biological heterogeneity.

The use of anthracyclines alone (which would be effective against the sarcomatous component) showed little or no effect on the carcinoma component (Rijswijk et al. 1994), but, conceptually, the addition of a sarcoma regimen to standard platinum-based chemotherapy could potentially add benefits, especially in those subtypes where the sarcomatous component is predominant or more aggressive (high grade and/or heterologous) as reported by Matsuo et al. (2016).

### Strengths and limitation

The main limitations of our study lie both on its retrospective nature and on the wide time frame during which the data were collected.

Nonetheless, this is one of the largest series reported in the literature specifically exploring the prognostic risk factors in early-stage UCSs.

Furthermore, the data were obtained from the collaboration of four leading academic institutions in Italy with similar management strategies and expert pathology evaluation.

### Future perspectives

The prognosis stratification based on histological characteristics should trigger future research both to clarify the exact pathogenesis and molecular biology of UCSs and to develop more targeted therapeutic regimens. Some authors claimed the hypothesis that heterologous UCSs are characterized by a higher EMT signature than homologous UCSs (Cherniack et al. 2017), so EMT targeting could be a promising therapeutic approach to improve the poor prognosis of these patients.

An ongoing phase Ib clinical study (NCT03206177), is investigating the feasibility of Galunisertib in combination with carboplatin/paclitaxel, assuming that TGF-β inhibitors can inhibit EMT and increase the proportion of the carcinoma component.

Furthermore, as suggested by Matsuo et al. (2016), another interesting pathogenic hypothesis that could occur in some UCSs, besides the more accredited EMT (Cantrell et al. 2015), is the inverse phenomenon, i.e., the mesenchymal–epithelial transition (MET).

Following the latter, agents with activity against sarcomas in combination with agents active against carcinomas may regain interest.

Future research addressing the issue of the therapeutic approach is certainly warranted, given also the enormous advances in the molecular field, where biological characterization of the tumor may indicate target mutations underlying neoplastic transformation.

## Conclusion

Early-stage UCSs are an aggressive and heterogeneous histological subtype of endometrial cancer, characterized by poor prognostic outcomes, despite the application of adjuvant therapy, thus suggesting that significant progress must be made in understanding the molecular landscape of these cancers. In our large series of UCSs, several histopathologic features as both carcinomatous and sarcomatous components, myometrial invasion and FIGO stage showed to play important prognostic roles. However, at multivariate analysis, only the sarcomatous component was confirmed to be a statistically significant negative prognostic factor. Although prospective clinical trials are warranted, they are difficult to design due to the rare nature of this disease. Collaborative approaches and multi-institutional studies are, therefore, advisable.

## Supplementary Information

Below is the link to the electronic supplementary material.

Supplementary file1 (DOCX 15 KB)

Supplementary file2 (JPG 237 KB)

Supplementary file3 (JPG 258 KB)

## Data Availability

Data are available if requested.
