# Supplementary material for: Is the sarcomatous component (homologous vs heterologous) the prognostic “driving force” in early-stage uterine carcinosarcomas? A retrospective multicenter study
Source: J Cancer Res Clin Oncol. 2023 Feb 11;149(9):6479–88. doi: 10.1007/s00432-023-04594-5 (PMC10356890; doi:10.1007/s00432-023-04594-5)
Supplement: Supplementary file 1 — Supplementary file1 (DOCX 15 KB) [file 432_2023_4594_MOESM1_ESM.docx]

**Table S1:** Post-operative and survival outcomes

| **Variables** |  | **Sarcomatous component** | |  |
| --- | --- | --- | --- | --- |
|  | **All**  **N (%)**  **N=95** | **Homologous/Group1**  **N (%)**  **N=60** | **Heterologous/Group2**  **N (%)**  **N=35** | **P-value**^†^ |
| **Post-operative outcomes** | | | | |
| **Hospital stay* (days)** | 4 (3-6) | 5 (3-7) | 4 (3-5) | 0.273**^‡^** |
| **Early post-operative complications** |  |  |  | 0.999**^§^** |
| No | 87 (91.6) | 55 (91.7) | 32 (91.4) |  |
| Yes | 8 (8.4) | 5 (8.3) | 3 (8.6) |  |
| **Adjuvant therapy** |  |  |  | 0.952 |
| No | 16 (16.8) | 10 (16.7) | 6 (17.1) |  |
| Yes | 79 (83.2) | 50 (83.3) | 29 (82.9) |  |
| **Type of adjuvant therapy** |  |  |  | 0.756 |
| BRT/EBRT | 14 (17.7) | 8 (16.0) | 6 (20.7) |  |
| CHT | 31 (39.2) | 19 (38.0) | 12 (15.2) |  |
| CHT-RT | 34 (43.0) | 23 (46.0) | 11 (37.9) |  |
| **Survival outcomes** | | | | |
| **Recurrences** | 50 (52.6) | 24 (40.0) | 26 (74.3) | **0.001** |
| **DOD** | 42 (44.2) | 21 (35.0) | 21 (60.0) | **0.018** |

*median (interquartile range)

**^‡^** U Mann-Whitney test

^§^ Fisher exact test

^†^ χ-squared test

BRT: brachytherapy, EBRT: external beam radiation therapy, CHT: chemotherapy, RT: radiotherapy, DOD death of disease
