# Supplementary figures and images for: Is the sarcomatous component (homologous vs heterologous) the prognostic “driving force” in early-stage uterine carcinosarcomas? A retrospective multicenter study
Source: J Cancer Res Clin Oncol. 2023 Feb 11;149(9):6479–88. doi: 10.1007/s00432-023-04594-5 (PMC10356890; doi:10.1007/s00432-023-04594-5)

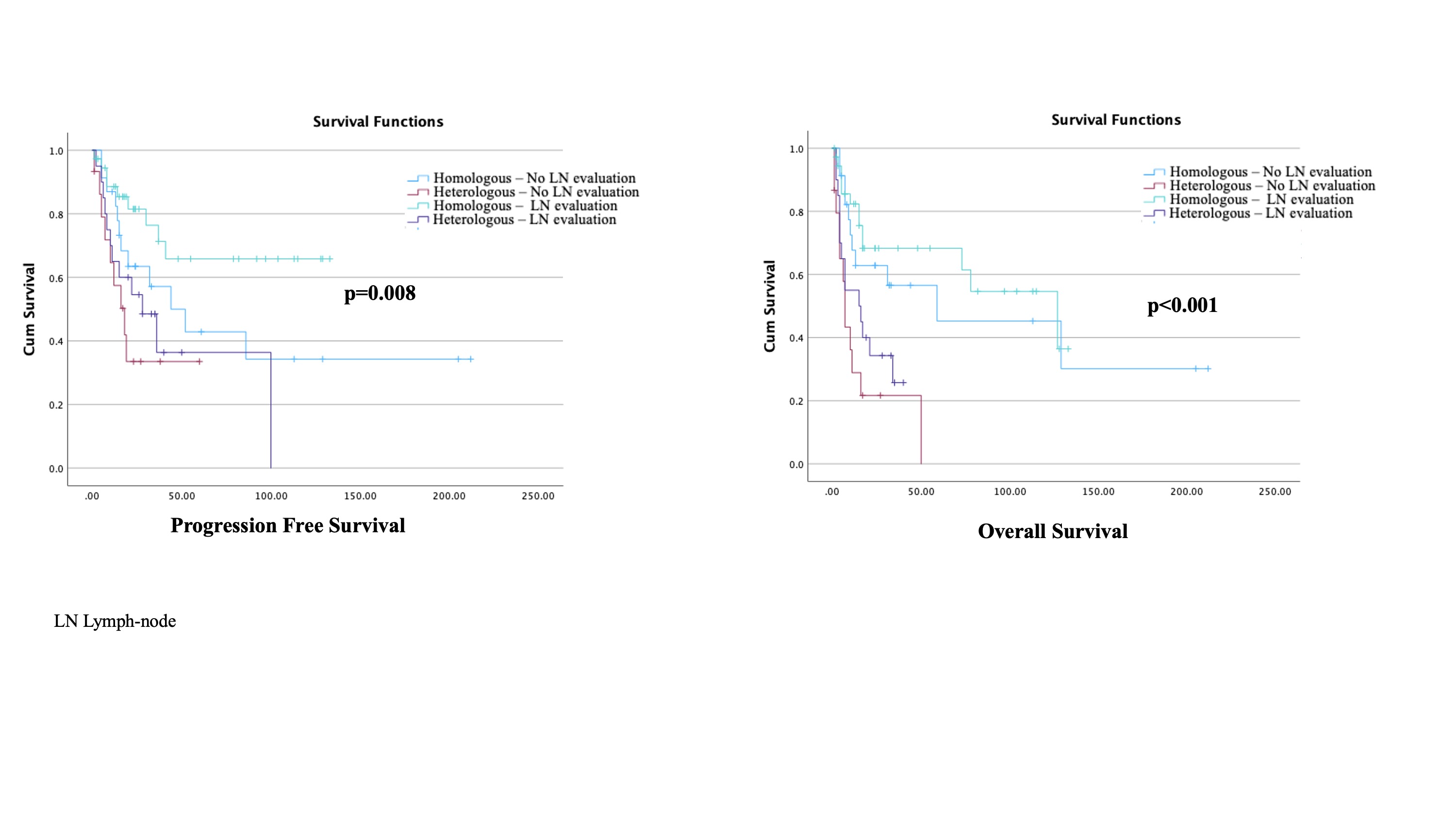

Supplement: Supplementary file 2 — Supplementary file2 (JPG 237 KB) [file 432_2023_4594_MOESM2_ESM.jpg]

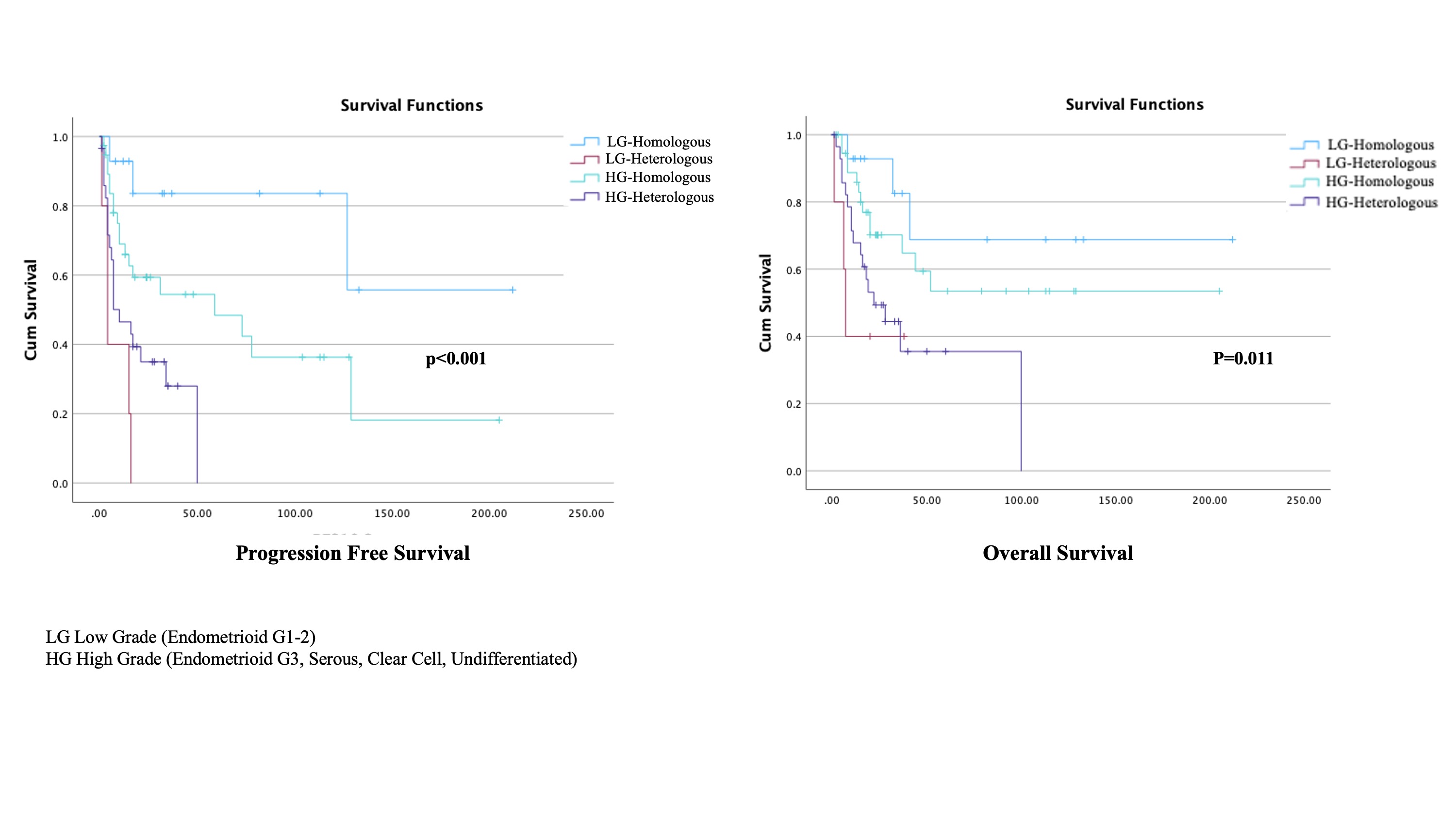

Supplement: Supplementary file 3 — Supplementary file3 (JPG 258 KB) [file 432_2023_4594_MOESM3_ESM.jpg]
